# Dietary Exposure to Aluminium and Health Risk Assessment in the Residents of Shenzhen, China

**DOI:** 10.1371/journal.pone.0089715

**Published:** 2014-03-03

**Authors:** Mei Yang, Lixin Jiang, Huiping Huang, Shengbo Zeng, Fen Qiu, Miao Yu, Xiaorong Li, Sheng Wei

**Affiliations:** 1 Department of Health Surveillance and Management, Futian District Center for Disease Control and Prevention of Shenzhen, Shenzhen, Guangdong, China; 2 Department of Laboratory, Futian District Center for Disease Control and Prevention of Shenzhen, Shenzhen, Guangdong, China; 3 Department of Epidemiology and Biostatistics, School of Public Health, Tongji Medical College of Huazhong University of Science and Technology, Wuhan, Hubei, China; The Ohio State University, United States of America

## Abstract

Although there are great changes of dietary in the past few decades in China, few are known about the aluminium exposure in Chinese diet. The aim of this study is to systematically evaluate the dietary aluminium intake level in residents of Shenzhen, China. A total of 853 persons from 244 household were investigated their diet by three days food records. Finally, 149 kinds of foods in 17 food groups were selected to be the most consumed foods. From them, 1399 food samples were collected from market to test aluminium concentration. High aluminium levels were found in jellyfish (median, 527.5 mg/kg), fried twisted cruller (median, 466.0 mg/kg), shell (median, 107.1 mg/kg). The Shenzhen residents' average dietary aluminium exposure was estimated at 1.263 mg/kg bw/week which is lower than the PTWI (provisional tolerable weekly intake). But 0–2 and 3–13 age groups have the highest aluminium intake exceeding the PTWI (3.356 mg/kg bw/week and 3.248 mg/kg bw/week) than other age groups. And the main dietary aluminium exposure sources are fried twisted cruller, leaf vegetables and bean products. Our study suggested that even three decades rapid economy development, children in Shenzhen still have high dietary aluminium exposure risk. How to control high dietary aluminium exposure still is a great public health challenge in Shenzhen, China.

## Introduction

Aluminium (Al) is the third most abundant element on earth. Aluminium can be toxic to bone, bone marrow and the nervous system [1]–[3]. There are proposed relationships between aluminium and the occurrence of neurodegenerative disorder, metabolic bone disease, dyslipemia and even genotoxic activity [4]–[6]. For example, Al accumulation in the brain can potentiate oxidative and inflammatory events, leading to tissue damage and play a key role in the Alzheimer's disease (AD) etiology [7], [8]. The subjects with the higher neonatal aluminium exposure had lower lumbar spine bone mass and lower hip bone mass which suggested the aluminium toxicity to bone development [9], [10]. Although food, water, airborne dust, antiperspirants, immunizations, allergy injections and antacids could be source of aluminium exposure for the general population, but food is the single largest contributor of aluminium intake [11]–[13]. Many foods or food products have been reported to have high level aluminium concentration, such as steamed pastry, bakery products (e.g. muffin, cake and pancake), tea, fried twisted cruller, drinking water, leavening product, shell, jellyfish and mung bean vermicelli [13]–[15]. The Joint Food and Agriculture Organization/World Health Organization Expert Committee on Food Additives (JECFA) established a provisional tolerable weekly intake (PTWI) for aluminium of 1 mg/kg body weight in 2006. Five years later, the committee re-evaluated the safety of aluminium and proposed a PTWI of 2 mg/kg body weight in June 2011. The PTWI applies to all aluminium compounds in food, including food additives. Unfortunately, many reports about the daily dietary aluminium exposures in various countries and regions showed that the aluminium exposure greatly exceeded the new safety reference level in these populations and consumption foods with aluminium-containing food additives were the major source of aluminium exposure for the general population [16]–[18].

Although the over intake of aluminium is essential to human health, but few investigations have been performed recently even the great change of daily dietary in the past thirty years in China. For example, Shenzhen city(2008 pop 8.77 million), the earliest economy special district of China economy reform, has experienced big changes of quantity and quality in residents dietary. But little is known about the dietary aluminium exposure level in Shenzhen residents. Here, we presented a latest study to assess the risk of dietary aluminium exposure in Shenzhen residents.

## Materials and Methods

### Ethics statement

Consent forms of all participants involved in the study were obtained. The study was approved by the Research Ethics Committee in Center for Disease Control and Prevention of Shenzhen, Guangdong, China.

### Food consumption data and food sampling

To identify major consumption foods in Shenzhen residents, 853 persons from 244 household were investigated to their dietary by three-stage cluster sampling as a part of the second individual and national food consumption survey in China in 2008 [19]. All persons were asked to complete a food record during three consecutive days to describe their daily dietary as precisely as possible. Portion sizes were estimated through photographs compiled in a manual. 853 residents reported to consume 564 kinds of foods during three consecutive days. Total 21,792 food products were recorded. The representative foods were selected based the results of food records. Only foods consumed proportions over 5% and more were selected. In addition, the foods known as having high aluminium concentration were also selected included in this study (even if its consumed proportions are below 5%). Finally, 149 kinds of foods (17, 566 food products) in 17 groups of food were selected to represent the most consumed foods of the whole diet of Shenzhen population. They were steamed pastry, bakery pastry, fried twisted cruller, fruits, milk, rice, eggs and egg products, meat, flour products (exclude ferment pastry), leaf vegetables, vegetables (exclude leaf vegetables), shell, fish, jellyfish, beverages, bean product and water.

The food sampling for all foods in 17 groups was performed from 2009 to 2012 by professional samplers. To take into account the potential regional variations in occurrence, the sampling was performed in food markets from six districts of Shenzhen city (Futian, Luohu, Nanshan, Baoan, Longgang and Yantian districts). A total of 1, 399 food samples in 149 kinds of foods were collected randomly from supermarket and farm products market. The food samples were put in aseptic food sampling bag and sent to laboratory as soon as possible.

### Aluminium concentrations analyses

Food samples were ground to powder in an IKA WERKE-M20 knife mill (IKA, Staufen, Germany) and dried in an oven at 85°C for 4 hours. Thereafter, they were stored in refrigerators at 4°C before test. A 0.5 g portion for inductively coupled plasma-mass spectrometry was accurately weighed and transferred into a capped PTFE vessel, then mixed with 3 ml of 65% nitric acid and 2 ml of 30% hydrogen peroxide. Digestion was carried out in a Milestone Ethos 1 microwave device (Sorisole, Italy). The temperature was ramped to 200°C within 10 min, following by a dwell-time of 15 min under microwave irradiation at 1000W. After completion, the vessel was taken out to cool completely at room temperature. The sample solution was transferred to a volumetric flask and diluted to 50 ml with ddH_2_O.

Aluminium in the sample was determined by inductively coupled plasma–mass spectrometry (Thermo XS series 2; Agilent, Lexington, MA, USA) according to the standard method of China [20]. Typical operating conditions and instrumental parameters employed in this study were: forward power 1357W; analogue detector 1784V; PC detector 3265V; sampling depth 135 mm; cool gas 13.0 l/min Ar; auxiliary gas 0.72 l/min Ar; extraction 125.5V. The detection limit (LOD) of aluminium was 0.12 µg/L. The linear range was from 10 µg/L to 2000 µg/L. And the recovery range was from 95.0% to 99.0%. According to international guidelines (GEMS/Food, 1995), the rate of non-detected values was lower than 60% and the non-detected values were assumed to be LOD/2.

### Exposure assessment

The individual food consumption data are from the food consumption survey of Shenzhen population in 2008. Mean consumption, 95th percentile (P95) and 99th percentile (P99) were calculated for each group of foods. Then average weekly food consumption data of per kg body weight was calculated for each group of food. Exposure to average weekly aluminium was assessed by multiplying the consumption data of per kg body weight and the median of aluminium concentration levels for each food group. Furthermore, Mean exposure, 95th percentile (P95) and 99th percentile (P99) were calculated for each group of food. Considering each of the 17 food groups, the mean contributions to total exposure to aluminium were calculated. Then the estimated average weekly exposure levels were compared with the PTWI. In addition, t test, Mann-Whinety U test, ANOVA and General Linear Model were used to compare the mean aluminium exposure levels by age and sex groups. All statistical analyses were conducted using the statistics program SPSS/PC version 12.0 (Chicago, IL,USA). All significance tests were 2-tailed, and *P* values less than 0.05 were considered to indicate statistical significance.

## Results

### Food consumption data

The results of mean consumption, 95th percentile (P95) and 99th percentile (P99) of individual consumption foods are summarized in Table 1. Among all 17 food groups, water was the most consumptive food with a mean of 1384.19 g/day, the second was fruit with a mean 234.10 g/day, and the next was beverage with a mean consumptive value of 233.38 g/day. The individual food consumptions per kg body weight were summarized in Table 1. Water was most consumptive food with a mean of 33.066 g/day per kg body weight (bw). The subsequences were milk (6.115 g), fruit (5.974 g) and beverage (5.217 g). Further stratified analyses by age and sex were shown in Table 2. The subjects in 0–2 year old group have most foods compared to other age groups (almost all *P*<0.05). Although there were no significant differences in food consumptions between females and males (all *P*<0.05), females tended to consume more fruits, milk, eggs, meat, fish, water and less beverages than males did.

**Table 1:** Food consumption data (mean, 95th and 99th percentiles) of the Shenzhen population.

Food group	Consumption data (g/day)			Consumption data (g/day bw)		
	Mean	P95	P99	Mean	P95	P99
Steamed pastry*	15.42±40.99	86.34	161.07	0.391±1.403	1.92	4.00
Bakery pastry	10.97±40.76	69.96	123.78	0.257±1.013	1.37	3.95
Fried twisted cruller	8.32±34.48	52.15	165.28	0.211±1.018	1.10	4.27
Fruits	234.10±237.71	671.66	906.44	5.974±9.076	20.33	43.05
Milk	162.87±307.19	750.00	1334.00	6.115±23.529	19.56	130.80
Rice	198.47±142.69	448.01	637.07	4.640±5.102	14.31	25.22
Eggs and egg products	38.79±58.09	106.64	154.04	0.970±1.789	3.06	8.84
Meat	166.07±103.05	346.08	444.74	3.854±4.146	10.39	20.87
Flour products	39.55±70.29	155.88	364.49	0.916±1.989	4.23	9.29
Leaf vegetables	119.09±147.36	355.73	758.69	3.056±6.406	9.82	25.86
Vegetables	205.11±204.56	606.96	993.39	5.035±7.086	16.00	36.12
Shell	0.79±7.35	0.00	21.20	0.016±0.139	0.00	0.56
Fish	90.56±103.87	286.50	457.80	2.215±3.680	7.58	18.23
Jellyfish	0.21±2.14	0.00	6.86	0.004±0.037	0.00	0.09
Beverages	233.38±653.73	1470.56	3456.34	5.217±18.933	29.92	68.17
Bean products	43.24±71.13	188.73	377.87	1.081±2.525	4.46	12.48
Water	1384.19±967.56	3019.00	3757.20	33.066±39.681	101.19	208.54

*including steamed stuffed bun, steamed bread, steamed twisted roll.

**exclude ferment pastry.

***exclude leaf vegetable.

**Table 2:** Food consumption data(g/day bw, mean, standard deviation) of the Shenzhen population by age, sex group.

Food group	0-		3-		14-		20-		40-		60-	
	Male	Female	Male	Female	Male	Female	Male	Female	Male	Female	Male	Female
Steamed pastry*	1.09±2.07	0.84±1.45	1.08±3.80	0.86±2.33	0.14±0.28	0.01±0.06	0.34±0.64	0.31±0.58	0.14±0.40	0.25±0.61	0.17±0.55	0.31±0.72
Bakery pastry	1.50±2.31	1.70±3.96	0.20±0.68	0.20±0.59	0.20±0.40	0.43±±0.66	0.22±0.56	0.26±1.32	0.09±0.26	010±0.32	0.26±0.66	0.19±0.62
Fried twisted cruller	0.22±0.74	0.56±1.49	0.47±1.74	0.87±2.61	0.03±0.12	0.14±0.34	0.21±0.74	0.20±0.89	0.02±0.12	0.04±0.20	0.02±0.13	0.16±0.56
Fruits	33.10±23.89	28.82±18.44	9.90±8.98	12.41±11.57	2.08±2.70	4.73±6.02	3.23±3.47	3.81±4.64	2.79±3.20	5.24±4.60	4.38±3.97	4.78±5.38
Milk	86.46±98.00	60.06±66.34	5.99±9.83	12.71±16.91	4.42±5.99	1.94±3.90	1.66±3.37	2.57±4.92	1.76±3.21	2.22±3.84	1.44±2.94	1.95±4.50
Rice	15.83±13.76	15.23±9.60	7.65±5.72	9.23±8.00	4.16±2.98	4.01±2.12	3.08±2.13	4.03±3.10	2.52±1.96	3.01±2.13	3.33±2.48	4.66±3.36
Eggs and egg products	5.38±4.60	4.31±3.66	1.27±1.41	1.78±2.69	0.73±0.50	0.79±0.56	0.57±0.54	0.83±1.02	0.34±0.40	0.80±2.39	0.70±0.77	0.80±0.71
Meat	13.98±11.41	13.70±8.36	6.24±4.93	7.06±6.34	3.81±2.12	3.92±2.83	2.85±1.55	3.57±1.97	2.16±1.47	2.55±1.56	1.60±1.04	2.54±1.70
Flour products**	2.47±3.93	0.58±1.08	2.22±3.74	2.27±4.00	0.92±0.94	1.06±1.52	0.55±1.12	0.63±1.19	0.66±1.52	0.70±1.05	0.48±0.68	0.66±0.87
Leaf vegetables	12.26±10.28	9.53±6.28	8.10±15.73	5.88±11.65	3.06±3.39	2.98±3.21	1.43±1.12	2.06±1.61	1.94±2.40	2.25±3.01	1.26±0.71	1.70±1.35
Vegetables***	16.44±11.42	19.50±13.01	10.15±11.58	10.15±12.62	4.17±4.16	4.97±5.16	3.14±3.07	4.17±4.78	2.29±2.02	2.61±2.49	4.05±3.80	5.16±5.01
Shell	0.08±0.20	0.05±0.17	0.00±0.00	0.00±0.00	0.01±0.06	0.03±0.10	0.00±0.03	0.03±0.25	0.00±0.01	0.01±0.02	0.01±0.07	0.06±0.20
Fish	8.43±9.99	9.81±7.12	4.03±6.30	4.71±5.51	1.66±1.98	1.47±1.78	1.17±1.32	1.73±1.89	1.18±1.37	1.78±2.09	1.55±1.77	1.64±1.78
Jellyfish	0.00±0.00	0.00±0.00	0.00±0.00	0.00±0.00	0.00±0.00	0.00±0.00	0.00±0.03	0.01±0.05	0.00±0.04	0.01±0.05	0.00±0.04	0.00±0.03
Beverages	17.27±36.40	7.88±14.98	9.85±49.09	5.53±11.34	9.12±17.11	8.68±14.96	4.14±10.45	3.44±9.87	2.02±7.23	6.01±16.84	5.67±14.20	3.77±9.67
Bean products	5.66±8.90	4.63±6.11	1.83±3.81	1.84±3.04	1.56±2.68	1.06±2.19	0.65±0.94	0.84±1.41	0.52±0.87	0.50±0.76	0.61±0.76	1.10±1.59
Water	141.45±94.88	152.67±77.00	44.46±53.43	52.28±53.46	20.94±13.51	27.48±23.15	22.22±15.68	27.34±20.01	23.92±14.66	26.19±15.92	16.05±14.32	29.18±23.38

*Include steamed stuffed bun, steamed bread, steamed twisted roll.

**exclude ferment pastry.

***exclude leaf vegetable.

### Concentrations of Aluminium in food

A total of 1399 food samples were analyzed for Aluminium concentration. The results were summarized in Table 3. Aluminium concentrations of 176 samples were lower than the limit of detection (LOD). Aluminium concentrations were varied considerably among the food samples (ranged from not detected (ND) to 1250.037 mg/kg). Among all 17 food groups, jellyfish has the highest aluminium concentration, ranging from 318.334 to 1000.359 mg/kg with a median of 527.500 mg/kg. The fried twisted cruller has the second highest aluminium level with the median was 466.000 mg/kg and range from 0.500 to 990.321 mg/kg. The third is shell with the median was 107.100 mg/kg and range from 6.611 to 292.894 mg/kg. Steamed pastry, baked pastry, leaf vegetables and bean products were found to contain the higher aluminium concentrations than fruits, rice, milk, meat and vegetables (exclude leaf vegetables).

**Table 3:** Concentrations of Aluminum in the 17 food groups in Shenzhen, China.

Food group	n	Al level (mg/kg)		range of aluminum levels(mg/kg)					
		range	median	ND#	0.01<	10.0<	50.0<	500<	≥500.01
Steamed pastry*	311	0.502–1226.016	17.110	-	112	53	16	110	20
Baked pastry	89	0.513–1250.037	12.000	-	42	22	8	15	2
Fried twisted cruller	34	0.500–990.321	466.000	-	7	4	-	7	16
Fruits	184	ND-7.789	0.005	94	90	-	-	-	-
Milk	25	ND-0.408	0.180	1	24	-	-	-	-
Rice	44	ND-31.446	0.821	6	36	2	-	-	-
Eggs and egg products	31	ND-0.560	0.219	8	23	-	-	-	-
Meat	70	ND-2.138	0.012	30	40	-	-	-	-
Flour products(exclude ferment pastry)	65	ND-41.247	4.000	3	48	14	-	-	-
Leaf vegetables	116	ND-210.753	13.560	1	48	54	13	-	-
Vegetables(exclude leaf vegetable)	108	ND-27.130	0.706	12	89	7	-	-	-
Shell	10	6.611–292.894	107.100	-	2	1	1	6	-
Fish	50	ND-44.329	0.664	11	33	6	-	-	-
Jellyfish	6	318.334–1000.359	527.500	-	-	-	-	3	3
Beverages	13	ND-0.213	0.010	6	7	-	-	-	-
Bean products	59	ND-143.003	10.610	1	28	26	3	1	-
Water	184	ND-0.118	0.052	3	181	-	-	-	-

*Include steamed stuffed bun, steamed bread, steamed twisted roll.

**exclude ferment pastry.

***exclude leaf vegetable.

# ND, Not detected.

### Dietary Aluminium exposure

Exposure to aluminium was assessed by multiplying the consumption data of per kg body weight and the median of aluminium concentrations in 17 food groups. Mean exposure, 95th percentile (P95) and 99th percentile (P99) were calculated for each group of food. The contribution of the total exposure to aluminium was calculated. The Shenzhen population's mean exposure to Aluminium was estimated at 1.263 mg/kg bw/week (Table 4). At the 95th percentile, exposure was estimated at 4.720 mg/kg bw/week. At the 99th percentile, exposure was estimated at 15.794 mg/kg bw/week. In the total dietary exposure, aluminium mainly came from fried twisted cruller, leaf vegetables and bean products. For the mean exposure level, the proportions from these three food groups were 54.60%, 22.98% and 6.34%, respectively. For the 95th percentile exposure level, the proportions from these three food groups respectively were 64.27%, 16.72% and 5.94%. And the contribution proportions from these three food groups respectively were 70.30%, 12.38% and 4.67% at the 99th percentile exposure level.

**Table 4:** Estimated dietary Aluminum exposures in a week (mean, 95th and 99th percentiles) of the Shenzhen population.

Food group	Dietary exposure (mg/kg bw/week)			Contribution of total exposure (%)		
	Mean±SD	P95	P99	Mean	P95	P99
Steamed pastry*	0.047±0.168	0.230	0.479	3.72	4.13	2.42
Baked pastry	0.022±0.085	0.115	0.332	1.74	2.06	1.67
Fried twisted cruller	0.689±3.322	3.582	13.94	54.60	64.27	70.30
Fruits	0.000±0.000	0.000	0.002	0.00	0.00	0.01
Milk	0.008±0.030	0.025	0.165	0.63	0.45	0.83
Rice	0.027±0.029	0.082	0.145	2.14	1.47	0.73
Eggs and egg products	0.001±0.003	0.005	0.014	0.08	0.09	0.07
Meat	0.000±0.000	0.000	0.002	0.00	0.00	0.01
Flour products**	0.026±0.056	0.118	0.260	2.06	2.12	1.31
Leaf vegetables	0.290±0.608	0.932	2.455	22.98	16.72	12.38
Vegetables***	0.025±0.035	0.079	0.178	1.98	1.42	0.90
Shell	0.012±0.104	0.000	0.418	0.95	0.00	2.11
Fish	0.010±0.017	0.035	0.085	0.79	0.63	0.43
Jellyfish	0.013±0.137	0.000	0.346	1.03	0.00	1.74
Beverages	0.000±0.001	0.002	0.005	0.00	0.04	0.03
Bean products	0.080±0.188	0.331	0.927	6.34	5.94	4.67
Water	0.012±0.014	0.037	0.076	0.95	0.66	0.38
Total	1.263	4.720	15.794	100.00	100.00	100.00

*Include steamed stuffed bun, steamed bread, steamed twisted roll.

**exclude ferment pastry.

***exclude leaf vegetable.

The aluminium exposure levels by age and sex in Shenzhen population are shown in Table 5. There is no significant different between aluminium exposure levels in females and males (*P* = 0.530), but it is significant among age groups (*P*<0.001). The 0–2 and 3–13 age groups have an excess aluminium intake than other groups and intake aluminium exceed the PTWI level (all *P*<0.05). Furthermore, there were no significant differences between aluminium exposure levels of males and females in most of age groups (most of *P*>0.05) except in age 60 and older group (*P* = 0.032). But females have more aluminium intake than males (1.335 vs. 1.185 mg/kg bw/week). The three main aluminium sources in dietary in each age group are shown in Table 6. The fried twisted cruller, leaf vegetables and bean products were the three most important sources of aluminium intake which accounted for 70%–85% of total aluminium intake in each age group.

**Table 5:** Dietary Aluminium exposures (mg/kg bw/week) in Shenzhen population by age and sex groups.

Age	Total		Male		Female		*P* *
	n	Mean±SD	n	Mean±SD	n	Mean ± SD	
0-	35	3.356±3.546	21	3.090±2.450	14	3.755±4.834	0.595
3-	121	3.248±7.210	70	2.788±5.854	51	3.881±8.761	0.412
14-	49	0.803±0.911	26	0.647±0.605	23	0.978±1.154	0.207
20-	340	1.065±2.732	147	1.024±2.426	193	1.096±2.950	0.809
40-	217	0.462±0.604	106	0.392±0.462	111	0.529±0.710	0.091
60-	91	0.720±1.446	38	0.386±0.486	53	0.959±1.819	0.032
Total	853	1.263±3.481	408	1.185±3.032	445	1.335±3.848	0.530

*t test or Mann-Whinety U test.

**Table 6:** The three main resource of dietary Aluminium exposure in Shenzhen residents by age.

Age group	Gender	Aluminium exposure*	Contribution of Aluminium exposure					
			Highest		Second Highest		Third Highest	
			Food group	Aluminium exposure*(%**)	Food group	Aluminium exposure*(%**)	Food group	Aluminium exposure*(%**)
0-	Male	3.090	Leaf vegetables	1.164(37.7)	Fried twisted cruller	0.736(23.8)	Bean products	0.421(13.6)
	Female	3.755	Fried twisted cruller	1.840(49.0)	Leaf vegetables	0.905(24.1)	Bean products	0.344(9.2)
3-	Male	2.788	Fried twisted cruller	1.535(55.1)	Leaf vegetables	0.769(27.6)	Bean products	0.136(4.8)
	Female	3.881	Fried twisted cruller	2.838(73.1)	Leaf vegetables	0.558(14.4)	Bean products	0.137(3.5)
14-	Male	0.647	Leaf vegetables	0.291(44.9)	Bean products	0.116(18.0)	Fried twisted cruller	0.105(16.2)
	Female	0.978	Fried twisted cruller	0.457(46.7)	Leaf vegetables	0.283(28.9)	Bean products	0.079(8.0)
20-	Male	1.024	Fried twisted cruller	0.699(68.3)	Leaf vegetables	0.136(13.3)	Bean products	0.049(4.8)
	Female	1.096	Fried twisted cruller	0.652(59.5)	Leaf vegetables	0.195(17.8)	Bean products	0.062(5.7)
40-	Male	0.392	Leaf vegetables	0.187(47.0)	Fried twisted cruller	0.067(17.1)	Bean products	0.039(9.9)
	Female	0.529	Leaf vegetables	0.214(40.4)	Fried twisted cruller	0144(27.3)	Bean products	0.037(7.0)
60-	Male	0.386	Leaf vegetables	0119(31.0)	Fried twisted cruller	0.069(17.8)	Bean products	0.045(11.8)
	Female	0.959	Fried twisted cruller	0.512(53.3)	Leaf vegetables	0.161(16.8)	Bean products	0.082(8.6)
Total	Male	1.185	Fried twisted cruller	0.584(49.2)	Leaf vegetables	0.318(26.9)	Bean products	0.084(7.1)
	Female	1.335	Fried twisted cruller	0.786(58.9)	Leaf vegetables	0.264(19.8)	Bean products	0.077(5.7)

* mg/kg bw/week.

** The proportion of Al intake of the food in the total dietary Al intake in the population.

## Discussion

The present study showed that the dietary aluminium exposure level in Shenzhen population is 1.263 mg/kg bw/week which is lower than the PTWI. The fried twisted cruller, leaf vegetables and bean products were major contributors to aluminium exposure in the population. Group analyses suggested that the children in Shenzhen intake high level aluminium over PTWI as while as females took more dietary aluminium than males.

Although rice is staple foods in Shenzhen, fried twisted cruller is a popular wheat flour food for breakfast, Cantonese morning tea and afternoon tea in Shenzhen, China. To get repaid rising in cooking fried twisted cruller, more aluminium-containing raising agents (for example, baking alum and baking soda) are usually used in fried twisted cruller which lead to high aluminium concentration in it. Except fried twisted cruller, aluminium-containing raising agents are also used in other steamed pastry and baked pastry which is a major dietary aluminium exposure resource. These findings are similar to the results from study in Hongkong, UK and USA, where dietary aluminium exposure is derived mainly from miscellaneous cereals and baking powder [13], [15], [18]. Therefore, to restrict aluminium-containing food additive using or use non-aluminium-containing food additive in food processing is a key way to reduce dietary aluminium exposure risk in Shenzhen population.

Mean dietary aluminium exposure from leaf vegetables was 0.290 mg/kg bw/week in Shenzhen. The median aluminium concentrations in leaf vegetable in Shenzhen was 13.560 mg/kg (ranged from ND to 210.753 mg/kg) were similar to Spanish (20.12 mg/Kg) but higher than that in French and UK [17], [21], [22]. Leaf vegetables could absorb metal from soil and accumulate during growth process [23], [24]. As the third distributed widely element in the earth crusted soil, aluminium could be absorbed by leaf vegetables through similar way. The main consumed leaf vegetables in Shenzhen are Chinese cabbage, green Chinese cabbage, water spinach and spinach. Among of them, spinach has the highest aluminium concentration, 45.838 mg/kg. Water spinach has the second highest aluminium concentration, 13.966 mg/kg and lettuce has the third highest aluminium concentration, 10.696 mg/kg. Considering the high consumption of these leaf vegetables in Shenzhen residents dietary, leaf vegetables were the second most important aluminium sources in this population.

Although aluminium intake level in adult did not exceed PTWI, but children in Shenzhen have high risk in aluminium exposure than adults even this city is the leading developed area in China. The mean aluminium exposure level in children in Shenzhen is 3.272 mg/kg bw/week which is higher than the PTWI level (2 mg/kg bw/week) and far higher than the mean aluminium intake level 0.44 mg/kg bw/week in French children [17]. In Shenzhen, Children over 3 years older in Shenzhen will be sent to day care center or kindergarten while children over 7 years older will be sent to elementary school. In there, their breakfast and lunch will be supplied by those institutions or bought by themselves. Since they generally only share food in a dinner with their parents in a weekday, it is reasonable that there are differences between the aluminum intake of children and their parents did. A study has compared the aluminium intake level in children and their parents and found that the average aluminium intakes between children and their parents are significantly different even they shared same foods [25]. It suggested that children and adults faced different risk of aluminium exposure even they shared same diet. Children are in higher risk of aluminium exposure than adult regarding the adverse effect of aluminium on the nervous system development [26], [27]. From the consumption data of children, we found the fried twisted cruller is also the major aluminium exposure source for children in Shenzhen. To protect children from high aluminium exposure, the fried twisted cruller should be removed from the diet of children in Shenzhen.

There some limitation for current study. First, study participants in dietary survey already knew about involvement in the study. That may bias the results for their performing might be better. Second, the results for children may be biased by over estimation since the food records for children were filled by their parents. Third, we use the median of aluminium concentration and the mean of consumption to estimate the dietary exposure. This is a simple point assessment for dietary aluminium exposure, which may not fully present the aluminium exposure level of Shenzhen population. Probability assessment method may be needed for further aluminium exposure assessment. Third, only three days dietary records were used to assess aluminium dietary level. Such diet assessment is limited for not considering the season change of diet in Shenzhen. Hence, dietary aluminium exposure estimation may not precisely reflect the individual true exposure level. More comprehensive dietary investigations are needed for future aluminium exposure assessment.

As far as we know, this study is the latest systematically assessment on the dietary aluminium exposure of Chinese. It suggested that even with three decades rapidly development, residents in Shenzhen still face high dietary Aluminium exposure, especially children. How to control high dietary aluminium exposure still is a great public health challenge in Shenzhen, China.
